# Association Between Metabolic Syndrome and Risk of Renal Cell Cancer: A Meta-Analysis

**DOI:** 10.3389/fonc.2022.928619

**Published:** 2022-06-27

**Authors:** Wurong Du, Kaibo Guo, Huimin Jin, Leitao Sun, Shanming Ruan, Qiaoling Song

**Affiliations:** ^1^ The First School of Clinical Medicine, Zhejiang Chinese Medical University, Hangzhou, China; ^2^ Department of Oncology, Affiliated Hangzhou First People’s Hospital, Zhejiang University School of Medicine, Hangzhou, China; ^3^ Department of Oncology, The Fourth School of Clinical Medicine, Zhejiang Chinese Medical University, Hangzhou, China; ^4^ Oncology Department, The Second Affiliated Hospital of Zhejiang Chinese Medical University (Xinhua Hospital of Zhejiang Province), Hangzhou, China; ^5^ Department of Medical Oncology, The First Affiliated Hospital of Zhejiang Chinese Medical University (Zhejiang Provincial Hospital of Traditional Chinese Medicine), Hangzhou, China; ^6^ Education Department, The First Affiliated Hospital of Zhejiang Chinese Medical University (Zhejiang Provincial Hospital of Traditional Chinese Medicine), Hangzhou, China

**Keywords:** metabolic syndrome, renal cell cancer, risk factor, obesity, meta-analysis

## Abstract

**Background:**

Metabolic syndrome (MetS) has been related to increased risks of a variety of cancers. However, the association between MetS and the risk of renal cell cancer (RCC) remains not fully determined. This meta-analysis was conducted to investigate whether MetS is independently associated with the risk of RCC in adults.

**Methods:**

Relevant observational studies were obtained by searching PubMed, Embase, Cochrane’s Library, and Web of Science databases. Study characteristics and outcome data were extracted independently by two authors. The random-effect model was used for meta-analysis considering the possible influence of between-study heterogeneity. Predefined subgroup analyses were used to evaluate the possible influences of study characteristics on the outcome.

**Results:**

Eight studies involving 10,601,006 participants contributed to the meta-analysis. Results showed that MetS was independently associated with a higher risk of RCC in adult population (risk ratio [RR]: 1.62, 95% confidence interval [CI]: 1.41 to 1.87, p<0.001; I^2 ^= 85%). Subgroup analyses showed consistent association in men (RR: 1.52, 95% CI: 1.23 to 1.89, p<0.001) and in women (RR: 1.71, 95% CI: 1.28 to 2.27, p<0.001), in Asians (RR: 1.51, 95% CI: 1.25 to 1.83, p<0.001) and in Caucasians (RR: 1.76, 95% CI: 1.46 to 2.12, p<0.001), and in community derived (RR: 1.56, 95% CI: 1.34 to 1.82, p<0.001) and non-community derived population (RR: 1.87, 95% CI: 1.71 to 2.04, p<0.001). Differences in study design or quality score also did not significantly affect the association (p for subgroup difference both >0.05).

**Conclusions:**

MetS may be independently associated with RCC in adult population.

## Introduction

Renal cell cancer (RCC) is a common malignancy of the urinary system (1). According to the statistics in 2018, approximately 400,000 cases of RCC were diagnosed annually among the global population, and about 175,000 patients died of RCC annually (2, 3). Moreover, the global incidence of RCC has risen gradually during the last decade (4). Despite of the development of imaging techniques for cancer screening, about 30% of patients with RCC are diagnosed at the advanced stages, and the prognosis of patients with RCC remains poor, particularly for those with advanced stages (5). Therefore, identification of high-risk population for the development RCC is important for the early diagnosis of the malignancy (6, 7).

Metabolic syndrome (MetS) is defined as a cluster of metabolic disorders including central obesity, insulin resistance, high blood pressure, and dyslipidemia (8). The prevalence of MetS is also continuously increased worldwide, especially in people from the developing countries (9). Pathophysiologically, people with MetS are characterized of chronic inflammatory response, which has been identified as a key mechanism of carcinogenesis (10, 11). Moreover, some components and concomitant metabolic or clinic-hematologic factors such as diabetes (12) and hyperhomocysteinemia (13) have been suggested to affect the progression of a variety of chronic diseases, including cancer. A previous meta-analysis in 2012 showed that MetS is associated with an increased risk of overall cancers (14). However, subsequent studies showed that the association between MetS and cancers may be different according to the site of the cancer (15). For example, MetS has been related to increased risks of colorectal cancer (16), esophageal cancer, pancreatic cancer (17), breast cancer (18), endometrial cancer (19), and prostate cancer (20), but not to the risks of lung cancer (21) and gastric cancer (22). Although some studies have also evaluated the association between MetS and RCC (23–30), the results were not consistent and it remains unknown whether MetS is independently associated with the incidence of RCC. Therefore, we performed a meta-analysis to comprehensively investigate the possible relationship between MetS and the risk of RCC in adult population.

## Methods

The Preferred Reporting Items for Systematic reviews and Meta-Analyses (PRISMA) Statement (31, 32) was followed in this systematic review and meta-analysis. The methods of analyzing and reporting of the meta-analysis were consistent with the Cochrane’s Handbook for Systematic Review and Meta-analysis (33).

### Database Search

We systematically searched the four electronic databases, including PubMed, Embase, Cochrane’s Library and Web of Science using the combined keywords: (1) “metabolic syndrome” OR “insulin resistance syndrome” OR “syndrome X”; (2) “renal” OR “kidney”; and (3) “cancer” OR “tumor” OR “carcinoma” OR “neoplasm” OR “adenoma” OR “malignancy”. We only considered studies in human subjects, and no restriction was applied to the publication language. The citation lists of the related articles were also screened manually for possible relevant studies. The date of final database search was January 10, 2022.

### Study Inclusion

The PICOS criteria were followed during the determination of the inclusion criteria.

P (patients): adult (aged 18 or above) participants without the diagnosis of cancer at baseline.
I (exposure): participants with the confirmed diagnosis of MetS.
C (control): patients without the confirmed diagnosis of MetS.
O (outcomes): relative risks for the incidence of RCC compared between adults with and without MetS.
S (study design): observational studies, including cohort studies, case-control studies, or cross-sectional studies, published as full-length articles.

For studies with potential overlapped population, the one with the largest sample size was selected for the meta-analysis. Reviews, meta-analyses, preclinical studies, studies that did not evaluate the influence of MetS, or studies that did not report the risk of RCC were excluded from the meta-analysis.

### Data Collection and Evaluation of Study Quality

Database search, data collection, and study quality evaluation were separately performed by two independent authors. If disagreements occurred, discussion with the corresponding author was indicated to reach the consensus. Data regarding the study information, patient characteristics, definitions of MetS, study periods, and methods for the validation of RCC diagnosis were collected. Evaluation of study quality was achieved by the Newcastle-Ottawa Scale (NOS) (34). This scale varies between 1 to 9 stars and assesses the quality of the observational studies with three domains, including selection of the patients, comparability between patients with and without exposure, and strategies for the validation of the outcomes.

### Statistical Methods

Risk ratio (RR) and the 95% confidence interval (CI) were used to indicate the association between MetS and RCC. If RRs with more than one model of multivariate regression analyses were reported, we collected the most adequately adjusted RR for subsequent analysis. The standard errors (SEs) of RRs were calculated from the data of 95% CIs or p values, and the RRs were logarithmically transformed to maintain a normal distribution (33). Between study heterogeneity was assessed by the Cochrane’s Q test and estimation of the I^2^ statistic (35). Typically, an I^2^ > 50% was considered as the indicator of significant between-study heterogeneity. To minimize the influence of heterogeneity, we used the random-effect model to pool the RR data of each study in a conservative manner (33). Sensitivity analysis by excluding one dataset at a time was performed to confirm the stability of the findings. A series of subgroup analyses were conducted to reveal the influences of study characteristics on the associations according to variables such as sex, ethnicity, and source of the population, study design, and the study quality scores. The publication bias was assessed by visual examination for the symmetry of the funnel plots and the Egger’s regression test. We used the RevMan (Version 5.1; Cochrane Collaboration, Oxford, UK) and Stata 12.0 software for the statistics of the meta-analysis.

## Results

### Identification of Related Studies

**Figure 1:**
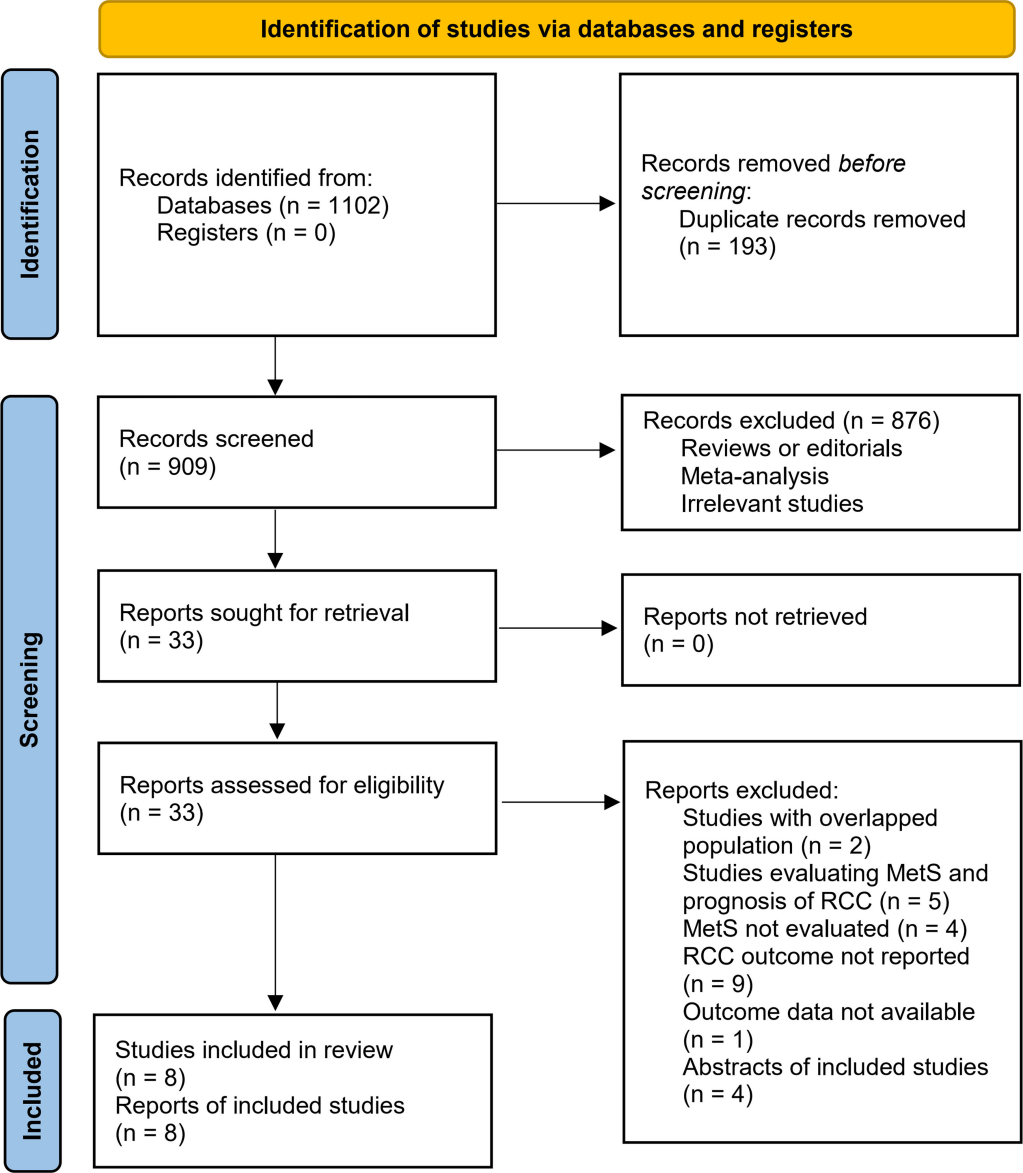
PRISMA diagram of literature search and study inclusion.

**Figure 1** summarizes the process of literature search. In brief, 1102 articles were retrieved in initial database search, and 193 duplications were subsequently excluded. Then, 33 articles were considered to be potentially relevant after excluding 876 irrelevant articles by title and abstract screening. In the final step of full-text review, another 25 studies were excluded according to the reasons listed in **Figure 1**. Finally, eight observational studies were identified and included in the meta-analysis (23–30).

### Summary of Study Characteristics

The characteristics of each study are displayed in **Table 1**. These studies were performed in Italy, the Netherlands, Norway, Austria, Sweden, Spain, Korea, and China, and designed as prospective cohorts (23–25, 28), retrospective cohorts (26, 27, 29), and the matched case-control study (30). Six of them included adult participants in community settings (23, 24, 26–28, 30), while the other two included adult patients with vascular diseases (25) and adults with hepatitis B virus infection (29). The sample sizes of the included studies varied between 6,172 and 7,613,865. The Adult Treatment Panel III-National Cholesterol Education Program (NCEP-ATP III) criteria were used for the diagnosis of MetS in all the included studies. Enrollment and follow-up of the participants were performed from 1994 to 2017. Overall, 13573 cases of RCC were observed in the meta-analysis, which were diagnosed and validated in national cancer registries (23–25) or codes of the International Classification of Diseases (ICD) (26–30). Confounding variables such as age, sex, smoking, alcohol intake, body mass index (BMI), and exercise etc. were controlled to a variable degree in the multivariate analyses. The NOS for the studies varied between seven and nine, indicating good study quality (**Table 2**).

**Table 1:** Characteristics of the included studies.

Study	Country	Study design	Population characteristics	Sample size	Definition of MetS	Follow-up period	Validation of RCC diagnosis	No. of patients with RCC	Variables adjusted	NOS
Russo 2008 (23)	Italy	PC	Community based population over 40 years	16,677	NCEP-ATP III	1999~2005	Local cancer registry	24	Age and sex	8
van Kruijsdijk 2013 (25)	the Netherlands	PC	Patients with vascular diseases	6,172	NCEP-ATP III	1996~2011	Netherlands Cancer Registry	24	Age, sex, smoking, and alcohol intake	8
Haggstrom 2013 (24)	Norway, Austria, and Sweden	PC	Community based population	560,388	NCEP-ATP III	1994~2006	National Cancer Registries	855	Age, sex, smoking, and BMI	9
Ko 2016 (26)	Korea	RC	Community based male population	61,758	NCEP-ATP III	2002~2013	ICD codes	87	Age, smoking status, alcohol intake, and exercise	7
Oh 2019 (27)	Korea	RC	Community based population over 20 years	7,613,865	NCEP-ATP III	2009~2017	ICD codes	3604	Age, sex, smoking, alcohol consumption, BMI, and regular physical exercise	7
Li 2020 (28)	China	PC	Community based male population	104,274	NCEP-ATP III	2006~2015	ICD codes	131	Age, education, income, smoking, and alcohol intake	9
Choe 2021 (29)	Korea	RC	HBV-infected adults over 40 years	1,504,880	NCEP-ATP III	2009~2016	ICD codes	2015	Age, sex, BMI, smoking status, alcohol consumption and physical activity	7
Lopez-Jimenez 2022 (30)	Spain	Matched C-C	Community based population over 40 years	732,992	NCEP-ATP III	2008~2017	ICD codes	6833	Age, sex, socioeconomic status, smoking status, and nationality	8

RCC, renal cell cancer; MetS, metabolic syndrome; NOS, Newcastle-Ottawa Scale; RC, retrospective cohort; PC, prospective cohort; C-C, case-control; HBV, hepatitis B virus; NCEP-ATP III, Adult Treatment Panel III-National Cholesterol Education Program; ICD, International Classification of Diseases; BMI, body mass index.

**Table 2:** Details of study quality evaluation *via* the Newcastle-Ottawa Scale.

Cohort studies	Representativeness of the exposed cohort	Selection of the non-exposed cohort	Ascertainment of exposure	Outcome not present at baseline	Control for age and sex	Control for other confounding factors	Assessment of outcome	Enough long follow-up duration	Adequacy of follow-up of cohorts	Total
Russo 2008 (23)	1	1	1	1	1	0	1	1	1	8
van Kruijsdijk 2013 (25)	0	1	1	1	1	1	1	1	1	8
Haggstrom 2013 (24)	1	1	1	1	1	1	1	1	1	9
Ko 2016 (26)	0	1	1	1	1	1	0	1	1	7
Oh 2019 (27)	0	1	1	1	1	1	0	1	1	7
Li 2020 (28)	1	1	1	1	1	1	1	1	1	9
Choe 2021 (29)	0	1	1	1	1	1	0	1	1	7
Case-control studies	Adequate definition of cases	Representativeness of cases	Selection of controls	Definition of controls	Control for age and sex	Control for other confounders	Exposure ascertainment	Same methods for events ascertainment	Non-response rates	Total
Lopez-Jimenez 2022 (30)	0	1	1	1	1	1	1	1	1	8

### Association Between MetS and RCC

**Figure 2:**
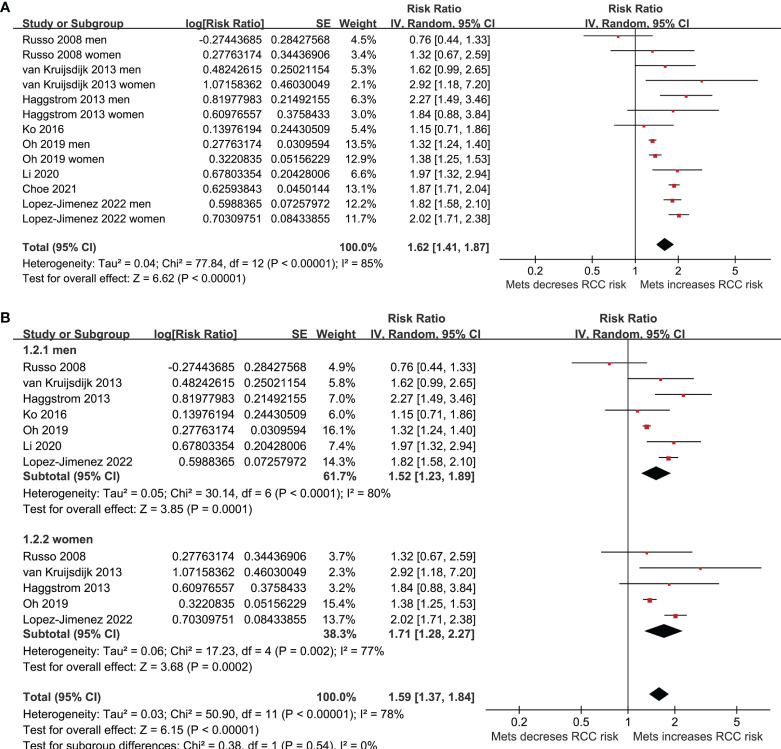
Forest plots for the meta-analysis of the association between MetS and RCC. **(A)**, overall meta-analysis; and **(B)**, group analysis according to the sex of the participants.

**Figure 3:**
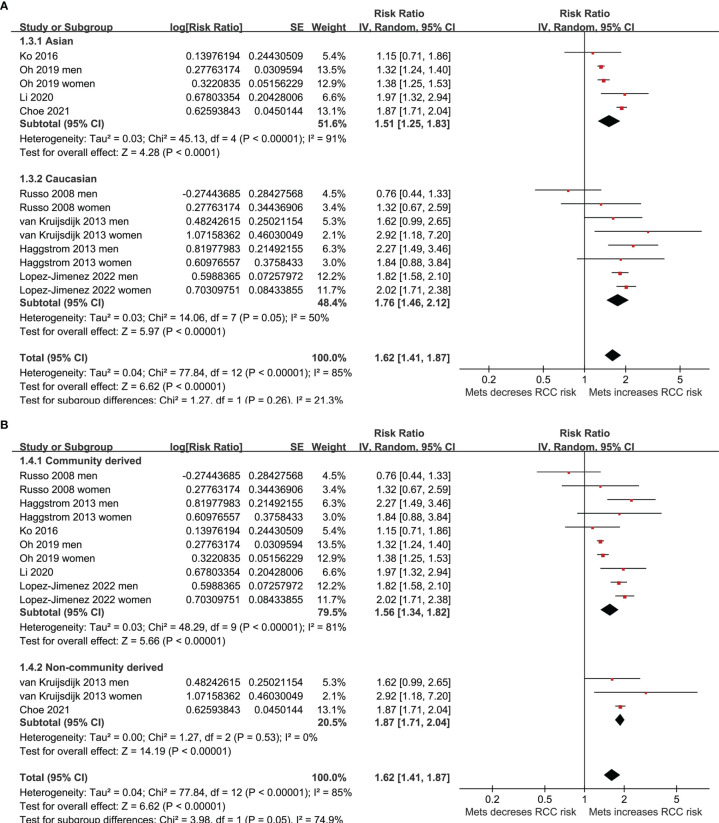
Forest plots for the meta-analysis of the association between MetS and RCC. **(A)**, subgroup analysis according to the ethnicity of the participants; and **(B)**, subgroup analysis according to the source of the participants.

**Figure 4:**
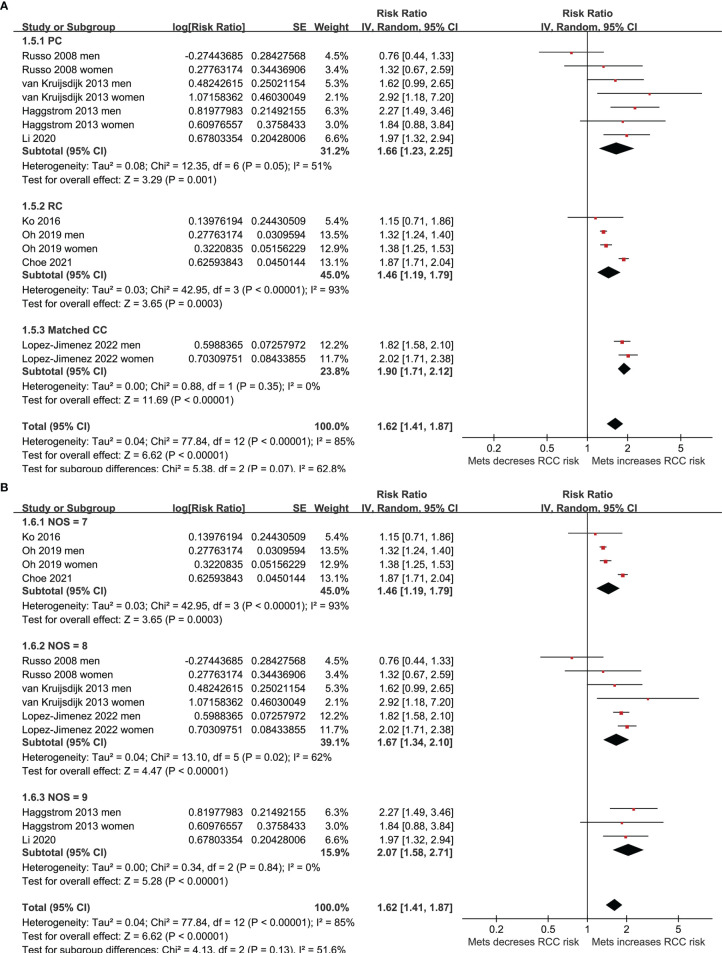
Forest plots for the meta-analysis of the association between MetS and RCC. **(A)**, subgroup analysis according to the study design; and **(B)**, subgroup analysis according to the study quality score.

Five of the included studies reported the association between MetS and RCC by sex of the included participants (23–25, 27, 30), and these datasets were included separately in the meta-analysis. Finally, 13 datasets from eight observational studies (23–30) were available for the meta-analysis. A significant between-study heterogeneity was observed (p for Cochrane’s Q test < 0.001, I^2^ = 85%). Pooled results with a random-effect model showed that MetS was independently associated with a higher risk of RCC in the adult population (RR: 1.62, 95% CI: 1.41 to 1.87, p<0.001; **Figure 2A**). Sensitivity analyses by omitting one dataset at a time showed similar results (RR: 1.58 to 1.68, p all <0.05). Predefined subgroup analysis showed a consistent association between MetS and RCC in men (RR: 1.52, 95% CI: 1.23 to 1.89, p<0.001) and in women (RR: 1.71, 95% CI: 1.28 to 2.27, p<0.001; p for subgroup difference=0.54; **Figure 2B**), in Asians (RR: 1.51, 95% CI: 1.25 to 1.83, p<0.001) and in Caucasians (RR: 1.76, 95% CI: 1.46 to 2.12, p<0.001; p for subgroup difference=0.26; **Figure 3A**), and in community derived (RR: 1.56, 95% CI: 1.34 to 1.82, p<0.001) and non-community derived population (RR: 1.87, 95% CI: 1.71 to 2.04, p<0.001; p for subgroup difference=0.05; **Figure 3B**). Moreover, a consistent association between MetS and RCC was also observed in subgroup analysis according to the study design and quality scores (p for subgroup difference both >0.05; **Figures 4A, B**).

### Publication Bias

**Figure 5:**
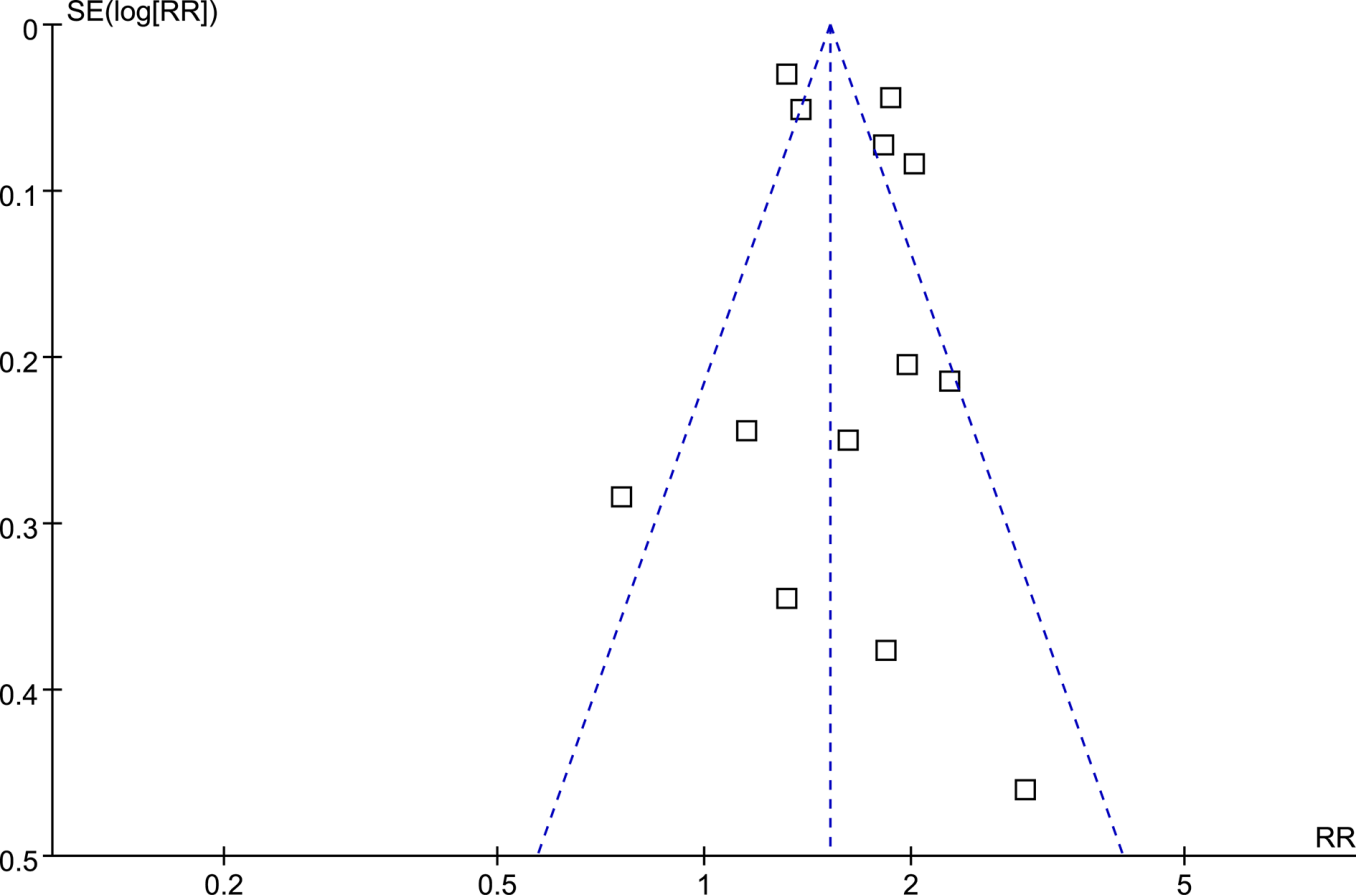
Funnel plots for the meta-analysis of the association between MetS and RCC.

Funnel plots for the association between MetS and RCC were symmetrical on visual examination (**Figure 5** **)**, suggesting low risk of publication biases, which were further confirmed by the results of Egger’s regression tests (p=0.57).

## Discussion

In this study, by pooling the results of the eight available observational studies, we found that MetS is independently associated with a higher risk of RCC in adult population. Further sensitivity analyses by excluding one dataset at a time did not significantly change the result, suggesting the stability of the results. Moreover, subgroup analyses showed consistent association between MetS and RCC in men and women, in Asians and Caucasians, in community derived and non-community derived population, in studies of different designs, and in studies with different quality scores. Taken together, results of this meta-analysis indicate that MetS may be an independent risk factor of RCC in adult population. These findings also suggest that people with MetS may be a high-risk population for RCC.

To the best of our knowledge, this may be the first meta-analysis which compressively evaluated the association between MetS and RCC. The strengths of the meta-analysis include extensive literature search, pooling the results of multivariate adjusted data, and conducting of multiple sensitivity and subgroup analyses to confirm the robustness of the finding. Collectively, the results of the meta-analysis support that MetS may be a risk factor for RCC. In this meta-analysis, we combined RR data that was adjusted most adequately to minimize the possible influences of confounding factors on the association between MetS and RCC, such as smoking status, alcohol intake, and exercise. This is important because it has been suggested that cigarette smoking is dose-dependently associated with the increased risk of RCC (36), while physical activity (37) and moderate alcohol consumption (38) are both inversely related to the incidence of RCC. Besides, consistent association between MetS and risk of RCC was retrieved in subgroup according to the sex and ethnicity of the participants. This is also important, because it has been suggested that the incidence of RCC is doubled in men than that in women (39), and a racial difference may also exist for the incidence of RCC (40). Besides, subgroup analyses according to source of the participants, study design, and study quality scores also showed consistent association between MetS and RCC in adult population, which further validated that MetS may be a risk factor for RCC.

There are some mechanisms underlying the association between MetS and the risk of RCC. Previous studies showed that people with MetS have reduced level of adiponectin, which may be related to obesity and insulin resistance (41). Lower circulating adiponectin has been involved in the pathogenesis of various obesity-related cancers, including RCC (42). Moreover, insulin resistance in people with MetS may be associated with the activation of the type 1 insulin-like growth factor (IGF-1) pathway (43). Because IGF-1 and insulin share overlapping downstream signaling pathways in normal and cancer cells, activation of the IGF-1 may promote malignant transformation promoting cell proliferation, dedifferentiation and inhibiting apoptosis, which have been involved in the pathogenesis of RCC (44). Moreover, metabolic disorders have also been associated with chronic systemic inflammation and oxidative stress, which have also been involved in the process of carcinogenesis, including the development of RCC (45, 46). Besides, some other mechanisms underlying the association between MetS and RCC have also been proposed, although to be validated in future studies. For example, changes of hormonal profile such as androgens in people with MetS may be involved in the pathogenesis of RCC (47–49). In addition, psychological factors including anxiety and stress have also been suggested as the mediators for the increased risk of RCC in people with MetS (50–52). Finally, the significant association between MetS and risk of RCC may also reflect the potential roles of the components of MetS as the risk factors of RCC, such as obesity (53), hypertension (54), and hyperglycemia (55).

The study also has some limitations. First, the NCEP-ATP III criteria were used to diagnose MetS in all of the included studies. Further studies are needed to determine if the association between MetS and the risk of RCC is consistent if MetS is diagnosed *via* other criteria, such as the International Diabetes Federation criteria. In addition, because of the observational nature of the included studies, there may be residual factors which may confound the association between MetS and RCC although multivariate adjusted data were pooled in this meta-analysis. Moreover, RCC is a heterogeneous cancer and the association between MetS and different pathological types of RCC should be evaluated in future studies. Finally, although a significant association between MetS and risk of RCC was retrieved, a causative relationship between MetS and RCC could not be derived from this study because observational studies were included. Future studies may be considered to determine whether reverse the metabolic disorders in people with MetS could reduce the risk of RCC.

## Conclusions

To sum up, results of the meta-analysis indicated that MetS is independently associated with the risk factor of RCC in adult population, which is consistent in men and women, and in Asians and Caucasians. People with MetS may be a high-risk population for RCC, and future studies are warranted to determine if reverse the metabolic disorders in this population could reduce the risk of RCC.

## Data Availability Statement

The original contributions presented in the study are included in the article/supplementary material. Further inquiries can be directed to the corresponding author.

## Author Contributions

WD and QS designed the study. WD and KG performed database search, study inclusion, quality evaluation, and data extraction. WD, HJ, LS, and SR performed statistical analyses and interpreted the data. WD drafted the manuscript. All authors critically reviewed the manuscript and approved its submission.

## Funding

This study was supported by Top Ten Thousand Talents Program of Zhejiang Province (SR, No. 2019-97), Zhejiang Provincial Program for the Cultivation of the Young and Middle-Aged Academic Leaders in Colleges and Universities (SR, No.2017-248), Zhejiang Provincial Project for the Key Discipline of Traditional Chinese Medicine (Yong Guo, No.2017-XK-A09) and Young Elite Scientists Sponsorship Program by CACM (No.2021-QNRC2-B13).

## Conflict of Interest

The authors declare that the research was conducted in the absence of any commercial or financial relationships that could be construed as a potential conflict of interest.

## Publisher’s Note

All claims expressed in this article are solely those of the authors and do not necessarily represent those of their affiliated organizations, or those of the publisher, the editors and the reviewers. Any product that may be evaluated in this article, or claim that may be made by its manufacturer, is not guaranteed or endorsed by the publisher.

## References

[1] Kanesvaran R Porta C Wong A Powles T Ng QS Schmidinger M . Pan-Asian Adapted ESMO Clinical Practice Guidelines for the Diagnosis, Treatment and Follow-Up of Patients With Renal Cell Carcinoma. ESMO Open (2021) 6(6):100304. doi: 10.1016/j.esmoop.2021.100304 34864348 PMC8645910

[2] Capitanio U Bensalah K Bex A Boorjian SA Bray F Coleman J . Epidemiology of Renal Cell Carcinoma. Eur Urol (2019) 75(1):74–84. doi: 10.1016/j.eururo.2018.08.036 30243799 PMC8397918

[3] Bray F Ferlay J Soerjomataram I Siegel RL Torre LA Jemal A . Global Cancer Statistics 2018: GLOBOCAN Estimates of Incidence and Mortality Worldwide for 36 Cancers in 185 Countries. CA Cancer J Clin (2018) 68(6):394–424. doi: 10.3322/caac.21492 30207593

[4] Gluba-Brzozka A Rysz J Lawinski J Franczyk B . Renal Cell Cancer and Obesity. Int J Mol Sci (2022) 23(6):3404. doi: 10.3390/ijms23063404 35328822 PMC8951303

[5] Chowdhury N Drake CG . Kidney Cancer: An Overview of Current Therapeutic Approaches. Urol Clin North Am (2020) 47(4):419–31. doi: 10.1016/j.ucl.2020.07.009 33008493

[6] Rossi SH Klatte T Usher-Smith J Stewart GD . Epidemiology and Screening for Renal Cancer. World J Urol (2018) 36(9):1341–53. doi: 10.1007/s00345-018-2286-7 PMC6105141 29610964

[7] Turajlic S Swanton C Boshoff C . Kidney Cancer: The Next Decade. J Exp Med (2018) 215(10):2477–9. doi: 10.1084/jem.20181617 PMC6170181 30217855

[8] Eckel RH Grundy SM Zimmet PZ . The Metabolic Syndrome. Lancet (2005) 365(9468):1415–28. doi: 10.1016/S0140-6736(05)66378-7 15836891

[9] Engin A . The Definition and Prevalence of Obesity and Metabolic Syndrome. Adv Exp Med Biol (2017) 960:1–17. doi: 10.1007/978-3-319-48382-5_1 28585193

[10] Fahed G Aoun L Bou Zerdan M Allam S Bouferraa Y Assi HI . Metabolic Syndrome: Updates on Pathophysiology and Management in 2021. Int J Mol Sci (2022) 23(2):786. doi: 10.3390/ijms23020786 35054972 PMC8775991

[11] Bishehsari F Voigt RM Keshavarzian A . Circadian Rhythms and the Gut Microbiota: From the Metabolic Syndrome to Cancer. Nat Rev Endocrinol (2020) 16(12):731–9. doi: 10.1038/s41574-020-00427-4 PMC8085809 33106657

[12] Ferro M Katalin MO Buonerba C Marian R Cantiello F Musi G . Type 2 Diabetes Mellitus Predicts Worse Outcomes in Patients With High-Grade T1 Bladder Cancer Receiving Bacillus Calmette-Guerin After Transurethral Resection of the Bladder Tumor. Urol Oncol (2020) 38(5):459–64. doi: 10.1016/j.urolonc.2020.02.016 32173242

[13] Giovannone R Busetto GM Antonini G De Cobelli O Ferro M Tricarico S . Hyperhomocysteinemia as an Early Predictor of Erectile Dysfunction: International Index of Erectile Function (IIEF) and Penile Doppler Ultrasound Correlation With Plasma Levels of Homocysteine. Med (Baltimore) (2015) 94(39):e1556. doi: 10.1097/MD.0000000000001556 PMC4616856 26426624

[14] Esposito K Chiodini P Colao A Lenzi A Giugliano D . Metabolic Syndrome and Risk of Cancer: A Systematic Review and Meta-Analysis. Diabetes Care (2012) 35(11):2402–11. doi: 10.2337/dc12-0336 PMC3476894 23093685

[15] Mili N Paschou SA Goulis DG Dimopoulos MA Lambrinoudaki I Psaltopoulou T . Obesity, Metabolic Syndrome, and Cancer: Pathophysiological and Therapeutic Associations. Endocrine (2021) 74(3):478–97. doi: 10.1007/s12020-021-02884-x 34625915

[16] Shen X Wang Y Zhao R Wan Q Wu Y Zhao L . Metabolic Syndrome and the Risk of Colorectal Cancer: A Systematic Review and Meta-Analysis. Int J Colorectal Dis (2021) 36(10):2215–25. doi: 10.1007/s00384-021-03974-y 34331119

[17] Rosato V Tavani A Bosetti C Pelucchi C Talamini R Polesel J . Metabolic Syndrome and Pancreatic Cancer Risk: A Case-Control Study in Italy and Meta-Analysis. Metabolism (2011) 60(10):1372–8. doi: 10.1016/j.metabol.2011.03.005 21550085

[18] Zhao P Xia N Zhang H Deng T . The Metabolic Syndrome Is a Risk Factor for Breast Cancer: A Systematic Review and Meta-Analysis. Obes Facts (2020) 13(4):384–96. doi: 10.1159/000507554 PMC7590763 32698183

[19] Wang L Du ZH Qiao JM Gao S . Association Between Metabolic Syndrome and Endometrial Cancer Risk: A Systematic Review and Meta-Analysis of Observational Studies. Aging (Albany NY) (2020) 12(10):9825–39. doi: 10.18632/aging.103247 PMC7288955 32439832

[20] Motterle G Luca DEZ Zecchini G Mandato FG Ferraioli G Bianco M . Metabolic Syndrome and Risk of Prostate Cancer: A Systematic Review and Meta-Analysis. Diabetol Metab Syndr (2020) 12:95. doi: 10.1186/s13098-020-00598-0 33133241 PMC7594475

[21] Qiao L Ma D Lv H Shi D Fei M Chen Y . Metabolic Syndrome and the Incidence of Lung Cancer: A Meta-Analysis of Cohort Studies. Diabetol Metab Syndr (2020) 12:95. doi: 10.1186/s13098-020-00598-0 33133241 PMC7594475

[22] Li Z Han H Chang Y . Association Between Metabolic Syndrome and the Incidence of Gastric Cancer: A Meta-Analysis of Cohort Studies. Diabetol Metab Syndr (2019) 11:83. doi: 10.1186/s13098-019-0478-y 31624504 PMC6785885

[23] Russo A Autelitano M Bisanti L . Metabolic Syndrome and Cancer Risk. Eur J Cancer (2008) 44(2):293–7. doi: 10.1016/j.ejca.2007.11.005 18055193

[24] Haggstrom C Rapp K Stocks T Manjer J Bjorge T Ulmer H . Metabolic Factors Associated With Risk of Renal Cell Carcinoma. PLoS One (2013) 8(2):e57475. doi: 10.1371/journal.pone.0057475 23468995 PMC3585341

[25] van Kruijsdijk RC van der Graaf Y Peeters PH Visseren FL . Cancer Risk in Patients With Manifest Vascular Disease: Effects of Smoking, Obesity, and Metabolic Syndrome. Cancer Epidemiol Biomarkers Prev (2013) 22(7):1267–77. doi: 10.1158/1055-9965.EPI-13-0090 23677576

[26] Ko S Yoon SJ Kim D Kim AR Kim EJ Seo HY . Metabolic Risk Profile and Cancer in Korean Men and Women. J Prev Med Public Health (2016) 49(3):143–52. doi: 10.3961/jpmph.16.021 PMC4898898 27255073

[27] Oh TR Han KD Choi HS Kim CS Bae EH Ma SK . Metabolic Syndrome Resolved Within Two Years is Still a Risk Factor for Kidney Cancer. J Clin Med (2019) 8(9):1329. doi: 10.3390/jcm8091329 PMC6780562 31466366

[28] Li X Li N Wen Y Lyu ZY Feng XS Wei LP . [Metabolic Syndrome Components and Renal Cell Cancer Risk in Chinese Males: A Population-Based Prospective Study]. Zhonghua Yu Fang Yi Xue Za Zhi (2020) 54(6):638–43. doi: 10.3760/cma.j.cn112150-20190711-00558 32842279

[29] Choe JW Hyun JJ Kim B Han KD . Influence of Metabolic Syndrome on Cancer Risk in HBV Carriers: A Nationwide Population Based Study Using the National Health Insurance Service Database. J Clin Med (2021) 10(11):2401. doi: 10.3390/jcm10112401 34072289 PMC8198770

[30] Lopez-Jimenez T Duarte-Salles T Plana-Ripoll O Recalde M Xavier-Cos F Puente D . Association Between Metabolic Syndrome and 13 Types of Cancer in Catalonia: A Matched Case-Control Study. PLoS One (2022) 17(3):e0264634. doi: 10.1371/journal.pone.0264634 35245317 PMC8896701

[31] Page MJ McKenzie JE Bossuyt PM Boutron I Hoffmann TC Mulrow CD . The PRISMA 2020 Statement: An Updated Guideline for Reporting Systematic Reviews. BMJ (2021) 372:n71. doi: 10.1136/bmj.n71 33782057 PMC8005924

[32] Page MJ Moher D Bossuyt PM Boutron I Hoffmann TC Mulrow CD . PRISMA 2020 Explanation and Elaboration: Updated Guidance and Exemplars for Reporting Systematic Reviews. BMJ (2021) 372:n160. doi: 10.1136/bmj.n160 33781993 PMC8005925

[33] Higgins J Thomas J Chandler J Cumpston M Li T Page M . Cochrane Handbook for Systematic Reviews of Interventions Version 6.2. London:The Cochrane Collaboration, (2021).

[34] Wells GA Shea B O'Connell D Peterson J Welch V Losos M . The Newcastle-Ottawa Scale (NOS) for Assessing the Quality of Nonrandomised Studies in Meta-Analyses. (2010).

[35] Higgins JP Thompson SG . Quantifying Heterogeneity in a Meta-Analysis. Stat Med (2002) 21(11):1539–58. doi: 10.1002/sim.1186 12111919

[36] Liu X Peveri G Bosetti C Bagnardi V Specchia C Gallus S . Dose-Response Relationships Between Cigarette Smoking and Kidney Cancer: A Systematic Review and Meta-Analysis. Crit Rev Oncol Hematol (2019) 142:86–93. doi: 10.1016/j.critrevonc.2019.07.019 31387065

[37] Behrens G Leitzmann MF . The Association Between Physical Activity and Renal Cancer: Systematic Review and Meta-Analysis. Br J Cancer (2013) 108(4):798–811. doi: 10.1038/bjc.2013.37 23412105 PMC3590672

[38] Bellocco R Pasquali E Rota M Bagnardi V Tramacere I Scotti L . Alcohol Drinking and Risk of Renal Cell Carcinoma: Results of a Meta-Analysis. Ann Oncol (2012) 23(9):2235–44. doi: 10.1093/annonc/mds022 22398178

[39] Scelo G Li P Chanudet E Muller DC . Variability of Sex Disparities in Cancer Incidence Over 30 Years: The Striking Case of Kidney Cancer. Eur Urol Focus (2018) 4(4):586–90. doi: 10.1016/j.euf.2017.01.006 28753845

[40] Sims JN Yedjou CG Abugri D Payton M Turner T Miele L . Racial Disparities and Preventive Measures to Renal Cell Carcinoma. Int J Environ Res Public Health (2018) 15(6):1089. doi: 10.3390/ijerph15061089 PMC6024978 29843394

[41] Ghadge AA Khaire AA Kuvalekar AA . Adiponectin: A Potential Therapeutic Target for Metabolic Syndrome. Cytokine Growth Factor Rev (2018) 39:151–8. doi: 10.1016/j.cytogfr.2018.01.004 29395659

[42] Fang J Xu X Mao Q Ying Y Zhang X Xie L . Lower Circulating Adiponectin is Associated With Higher Risk of Renal Cell Carcinoma: A Meta-Analysis. Int J Biol Markers (2020) 35(1):57–64. doi: 10.1177/1724600819898696 31973613

[43] Arai Y Kojima T Takayama M Hirose N . The Metabolic Syndrome, IGF-1, and Insulin Action. Mol Cell Endocrinol (2009) 299(1):124–8. doi: 10.1016/j.mce.2008.07.002 18672019

[44] Tracz AF Szczylik C Porta C Czarnecka AM . Insulin-Like Growth Factor-1 Signaling in Renal Cell Carcinoma. BMC Cancer (2016) 16:453. doi: 10.1186/s12885-016-2437-4 27405474 PMC4942928

[45] Reuter S Gupta SC Chaturvedi MM Aggarwal BB . Oxidative Stress, Inflammation, and Cancer: How are They Linked? Free Radic Biol Med (2010) 49(11):1603–16. doi: 10.1016/j.freeradbiomed.2010.09.006 PMC2990475 20840865

[46] Zhang GM Zhu Y Ye DW . Metabolic Syndrome and Renal Cell Carcinoma. World J Surg Oncol (2014) 12:236. doi: 10.1186/1477-7819-12-236 25069390 PMC4118156

[47] Salciccia S Del Giudice F Gentile V Mastroianni CM Pasculli P Di Lascio G . Interplay Between Male Testosterone Levels and the Risk for Subsequent Invasive Respiratory Assistance Among COVID-19 Patients at Hospital Admission. Endocrine (2020) 70(2):206–10. doi: 10.1007/s12020-020-02515-x PMC7543668 33030665

[48] Kalyani RR Dobs AS . Androgen Deficiency, Diabetes, and the Metabolic Syndrome in Men. Curr Opin Endocrinol Diabetes Obes (2007) 14(3):226–34. doi: 10.1097/MED.0b013e32814db856 17940444

[49] Pak S Kim W Kim Y Song C Ahn H . Dihydrotestosterone Promotes Kidney Cancer Cell Proliferation by Activating the STAT5 Pathway *via* Androgen and Glucocorticoid Receptors. J Cancer Res Clin Oncol (2019) 145(9):2293–301. doi: 10.1007/s00432-019-02993-1 PMC11810384 31401673

[50] Hoffmann MS Brunoni AR Stringaris A Viana MC Lotufo PA Bensenor IM . Common and Specific Aspects of Anxiety and Depression and the Metabolic Syndrome. J Psychiatr Res (2021) 137:117–25. doi: 10.1016/j.jpsychires.2021.02.052 33677215

[51] Maggi M Gentilucci A Salciccia S Gatto A Gentile V Colarieti A . Psychological Impact of Different Primary Treatments for Prostate Cancer: A Critical Analysis. Andrologia (2019) 51(1):e13157. doi: 10.1111/and.13157 30281167

[52] Wang YH Li JQ Shi JF Que JY Liu JJ Lappin JM . Depression and Anxiety in Relation to Cancer Incidence and Mortality: A Systematic Review and Meta-Analysis of Cohort Studies. Mol Psychiatry (2020) 25(7):1487–99. doi: 10.1038/s41380-019-0595-x 31745237

[53] Gild P Ehdaie B Kluth LA . Effect of Obesity on Bladder Cancer and Renal Cell Carcinoma Incidence and Survival. Curr Opin Urol (2017) 27(5):409–14. doi: 10.1097/MOU.0000000000000425 28650865

[54] Colt JS Schwartz K Graubard BI Davis F Ruterbusch J DiGaetano R . Hypertension and Risk of Renal Cell Carcinoma Among White and Black Americans. Epidemiology (2011) 22(6):797–804. doi: 10.1097/EDE.0b013e3182300720 21881515 PMC3188386

[55] Joh HK Willett WC Cho E . Type 2 Diabetes and the Risk of Renal Cell Cancer in Women. Diabetes Care (2011) 34(7):1552–6. doi: 10.2337/dc11-0132 PMC3120193 21602426

